# Interruption of the Gut Integrity Contributes to Early Accumulation of Amyloid-β in the Enteric Nervous System in Rats Supplemented with a High-Fat Diet

**DOI:** 10.1007/s12035-025-05261-1

**Published:** 2025-08-08

**Authors:** Zeinab Gawish, Maha Gamal, Dalia Azmy Elberry, Esraa A. Hegazy, Laila Ahmed Rashed, Sara Adel Hosny, Marwa Nagi Mehesen, Asmaa Mohammed ShamsEldeen

**Affiliations:** 1 Department of Medical Physiology, Faculty of Medicine, Cairo University, Cairo, Egypt; 2 Department of Microbiology, Faculty of Medicine, Cairo University, Cairo, Egypt; 3 Department of Histology and Cell Biology, Faculty of Medicine, Cairo University, Cairo, Egypt; 4 Department of Biochemistry and Molecular Biology, Faculty of Medicine, Cairo University, Cairo, Egypt; 5 Department of Pharmacology, Faculty of Medicine, Cairo University, Cairo, Egypt

**Keywords:** High-Fat Diet, Enteric Plexus, Amyloid, Oxidative Stress, GFAP

## Abstract

Consumption of a high-fat diet (HFD) contributes to numerous chronic illnesses, including neurological disorders and gastrointestinal dysfunction. The study design included four groups, each consisting of six rats: the control group was fed regular chow, while groups 2 (HFD 2W), 3 (HFD 4W), and 4 (HFD 8W) were given a 60% HFD for 2, 4, and 8 weeks, respectively. A significant change in the latency to the platform in the water maze, a decrease in the percentage of successful cycles in the Y-maze, and a reduction in the percentage of time spent with the novel object were observed between the baseline and endpoint results in the HFD 4W and HFD 8W groups; notably, this was not the case in the control and HFD 2W groups. Consistent with the increased duration of HFD intake, marked damage to the cortical pyramidal cells as well as the mucosa of the ileum and colon was recorded. The optical density of amyloid deposition was significantly increased in the myenteric plexus before the cerebral cortex. In conclusion, increasing the duration of 60% HFD consumption was associated with significant deterioration of spatial and working memory, an increase in lipid profile, and amyloid deposition in the enteric nervous system, which began even before the observable involvement of the cerebral cortex.

**Graphical Abstract:**

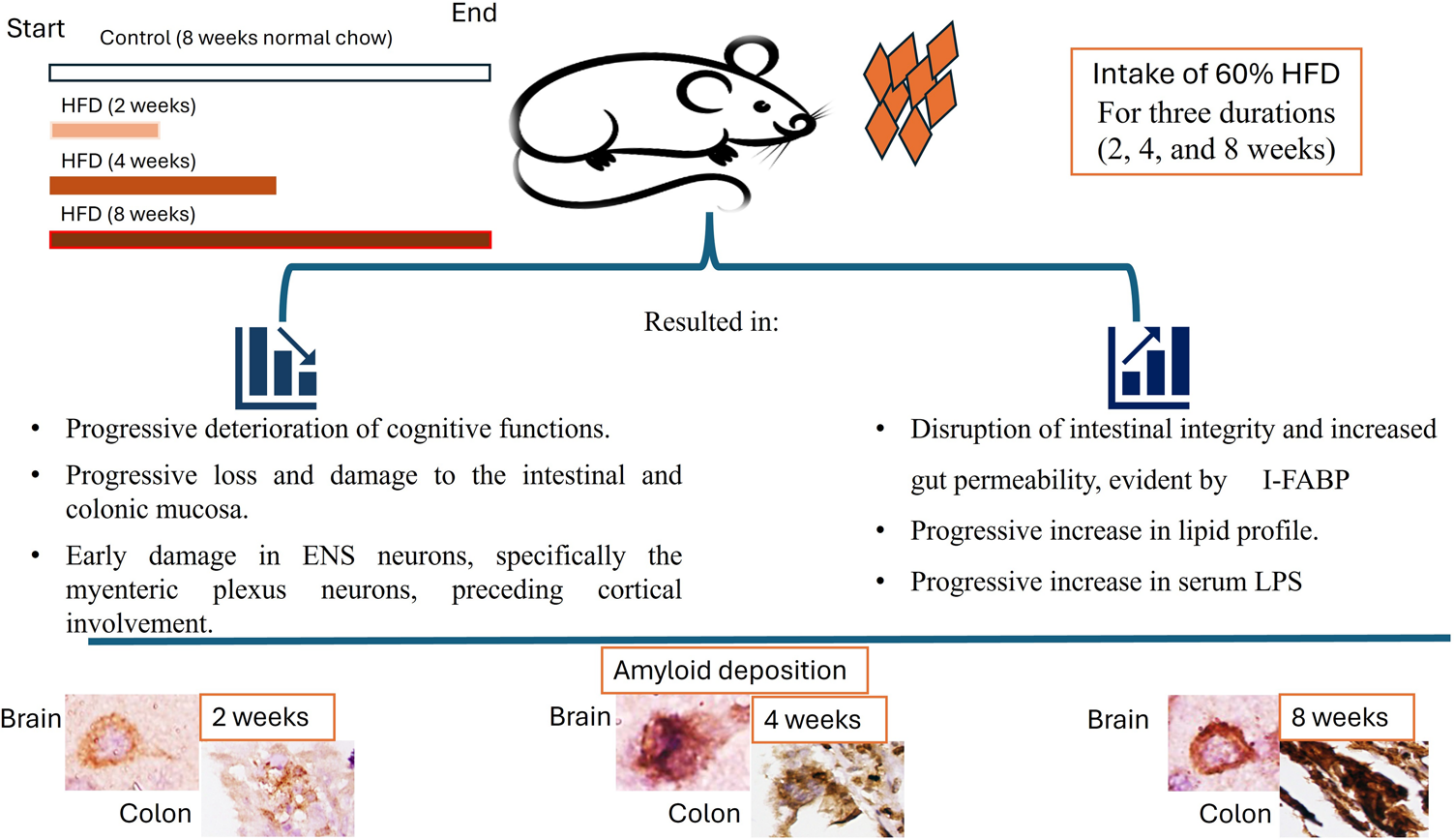

**Supplementary Information:**

The online version contains supplementary material available at 10.1007/s12035-025-05261-1.

## Introduction

High-fat diet (HFD) intake and consequent obesity contribute to the development of several chronic illnesses, such as type 2 diabetes mellitus, cardiovascular diseases, and several neurological disorders [1]. The steady rise in the prevalence of obesity has gained attention worldwide and raised significant economic and health concerns [2]. The intake of HFD, which represents a well-established model of a high-caloric diet, can increase fat mass and induce gastrointestinal dysfunction (GID) [3].

Alteration involving local oxidative stress and excessive production of reactive oxygen species (ROS) may disrupt local enteric neuronal homeostasis, affecting neuronal density, neuromuscular transmission, ganglia size, and the expression of glial markers [4]. Consumption of a 42–60% HFD increases brain beta-amyloid (Aβ) deposition levels, a hallmark of Alzheimer's disease (AD), and contributes to gliosis in APP/PS1 mice; however, these changes likely depend on the time frame of HFD consumption [5].

The enteric nervous system (ENS) is organized into two major plexuses: the myenteric and the submucosal plexus [6]. Although the ENS functions autonomously, its innervation connections with the central nervous system (CNS) are maintained through the vagus nerve and the prevertebral ganglia [7].

In this context, emerging studies suggest that GID, dysbiosis, and enteric neuropathy may increase the risk of developing AD [8]. Liu and colleagues'study demonstrated that intracolonic administration of Aβ42 induced local ENS dysfunction and brain gliosis in a mouse model of β-amyloidosis [9].

The intestinal tract is the body's largest barrier, promoting internal homeostasis, absorbing dietary nutrients, and protecting against external insults. The intestinal barrier comprises the gut microbiota, mucus, mucosal cell layer, tight junctions, and immune cells. Every component of this barrier is susceptible to modification by external factors such as dietary fat [10].

The intestinal fatty acid-binding protein (I-FABP) is expressed in the mucosal layer of the small intestine. HFD disrupts the integrity of the gut barrier, leading to increased intestinal permeability and the subsequent release of I-FABP, resulting in a rise in its plasma concentration [11].

The disruption of intestinal integrity and increased gut permeability is associated with high serum levels of I-FABP. The latter is associated with type 1 diabetes and diabetic nephropathy [12], chronic heart failure-mediated cardiac dysfunctions [13], and is correlated with coronary artery stenosis in type 1 diabetic patients [11]. Moreover, I-FABP may serve as a predictive biomarker of neurological complications associated with intestinal damage [14].

Therefore, we aimed to investigate the time course effect of HFD consumption at three durations over eight weeks on gut barrier integrity by measuring the levels of I-FABP in animals exposed to HFD for two, four, and eight weeks. Moreover, we investigated alterations in enteric neuronal homeostasis, as indicated by the early development of enteric amyloidosis, as well as changes in cognitive function among different groups of animals exposed to a 60% HFD for varying durations.

## Materials and Methods:

### Acclimatization and Ethical Approval

Twenty-four adult male Sprague–Dawley (SD) rats weighing between 160 and 180 g were used in this study. During the entire experimental period, the animals were acclimatized for 7 days to normal environmental conditions, including a dark/light cycle (8:00 am-8:00 pm), temperature (22–25 °C), and relative humidity (60%). Baseline assessments of body weight and cognitive functions were conducted for all rats. The animals were housed in the animal facility at Cairo University's Faculty of Medicine.

All experimental procedures were performed following the"Institutional Animal Care and Use Committee"at Cairo University (CU-IACUC); IACUC Protocol Number: CU III-F-20–23.

### Experimental Design and Animal Study

The animals were randomly classified into four groups (six rats each). Group 1 (the control group) received regular rat chow (10% kcal fat) [11]. Group 2 (HFD 2 W) was fed HFD (60% kcal fat) for 2 weeks and then sacrificed. Group 3 (HFD 4 W) received HFD (60% kcal fat) for 4 weeks before sacrifice. Group 4 (HFD 8 W) received HFD (60% kcal fat) for 8 weeks and was then sacrificed. Thus, we present the first report investigating the time-dependent effect of consuming 60% HFD.

### Diet Composition

The rats received a rodent control diet containing 10% of their total calories from fat (D12450Ji, Research Diets) or a diet with 60% of their total calories from fat (Research Diets D12492) [15]. Each group of rats, either the control or the HFD group, received the control diet, which consisted of 10% kcal from fat, or the diet with 60% kcal from fat throughout the study until the time of sacrifice. The rat chow was provided ad libitum, and all rats had access to water (Supplementary File).

At the end of each time point, the rats were assessed for body weight changes and cognitive function. The rats in the control group were sacrificed at the end of the experiment after 8 weeks.

### Evaluation of Cognitive Function

#### Morris Water Maze Test for Testing the Spatial Memory

The Morris task [16] was conducted in a water tank (150 cm in diameter) filled to a depth of 30 cm. The water was rendered opaque by adding approximately 0.5 L of dried milk powder and maintained at 26 ± 1 °C. The rats underwent four trials per day for four consecutive days. The platform was submerged 1.5 cm below the water surface. Twenty-four hours after the last acquisition trial (on the fifth day), a probe trial was conducted to assess the rats'spatial memory of the submerged platform's location.

Acquisition of the platform location was assessed during the first four days. Each trial began by placing a rat into the water facing the tank wall. Each starting position (north, south, east, and west) was used once per day in a randomized order across the four trials. Each trial ended when the rat climbed onto the platform or after 2 min had elapsed. Once on the platform, the rat remained there for 30 s. After completing the fourth trial, rats were gently dried with a towel and returned to their home cages. The inter-trial interval was consistently maintained at 30 min.

During the probe trial, the platform was removed, and the rat was allowed to search the pool for 60 s before it was removed. During this period, rats were expected to spend more time searching in the quadrant where the platform had previously been located compared to the other three quadrants [16].

Standard protocols for the Morris water maze (acquisition and probe trials) were employed to assess spatial learning and reference memory, which are indicators of long-term memory in animals [17, 18]. The latency to the platform, the time each rat required to reach the platform, and the percentage of time spent in the target quadrant were recorded and analyzed using a video camera to detect latency to the platform [19].

#### The Y-Maze Spontaneous Alternation Test for the Evaluation of Working Memory

For this behavioral test, a wooden Y-maze apparatus was used, consisting of three arms of equal size (40 cm long × 15 cm wide × 35 cm high) extending from the center at 120° [20]. Each rat was placed in the maze for 5 min, starting from the center, and the sequence of arm choices was recorded. A successful cycle was defined as the rat choosing three successive different arms. The percentage of successful cycles was measured [21].

#### Modified Novel Object Test for the Evaluation of Episodic-Like Memory

We followed our previous protocol for recording novel object investigation time [22]. This test assesses learning and recognition memory. Each animal was allowed to explore the arena freely for 10 min to acclimate, then returned to its cage for 20 min of rest. The area was cleaned with 70% ethanol after each exploration. Two different objects were then placed diagonally in two corners, and each rat was allowed to explore the area freely for 10 min before returning to its cage for another 20 min of rest. Finally, one of the two objects was replaced with a novel object, and each rat was allowed to re-explore the area for 10 min. The animals were monitored using a video camera, and the total investigation time of the novel object was calculated for each rat. The following equation was used to calculate the percentage of total investigation time:

$$Percentage of total investigation time=\frac{Time with novel location or object}{Time with novel location or object+Time with familiar location or object}x 100$$

At the end of the study, under general anesthesia induced by intraperitoneal injection of pentobarbital sodium (50 mg/kg) [23]. Blood samples were collected from each rat by cardiac puncture after thoracotomy, followed by euthanasia via decapitation. Ten milliliters of blood were obtained from each rat, and the blood was centrifuged at 3000 rpm for 15 min to collect the serum, which was then stored at −20 °C until analysis.

### Biochemical Evaluation

Blood samples were collected, and serum from all groups was used to estimate lipid profiles, Lipopolysaccharide (LPS) and I-FABP levels.

#### Lipid Profile

Estimation of lipid profiles **(cholesterol and triglycerides)** was done using the total cholesterol rat ELISA kit (Competitive ELISA, Cat No. MBS722885) and the rat triglyceride (TG) ELISA kit (Competitive ELISA, Cat No. MBS726298), respectively.

Briefly, 100 μL of the sample was added to coated wells and shaken, followed by the addition of 50 μL of conjugate mixed with the sample in each well, except for the control. Wells were covered and incubated for 1 h at 37 °C. After washing and removing liquid, substrates A and B were added, followed by a stop solution. Total cholesterol and triglyceride levels were measured at an optical density (O.D.) of 450 nm using a microplate reader.

#### Lipopolysaccharide (LPS)

Lipopolysaccharide (LPS) level was estimated using the rat LPS ELISA kit (Cat No. MBS268498). After adding LPS standards and samples to the corresponding wells, 100 μL of biotinylated antibody was added to each well. Wells were sealed and incubated at 37 °C for 1 h. Then, 100 μL of prepared enzyme conjugate was added to each well, followed by incubation at the same temperature for 30 min. Subsequently, 100 μL of prepared color reagent was added to each well and incubated until the highest standards exhibited a darker coloration and a color gradient appeared. Finally, 100 μL of color reagent C was added to each well, and results were read at an O.D. of 450 nm [24].

#### I-FABP

I-FABP level was estimated using the rat I-FABP ELISA kit (Cat No. MBS164325). 50 μL of the standard was added to the standard wells, and 40 μL of the sample was added to each well with 10 μL of anti-I-FABP antibody. Then, 50 μL of streptavidin-HRP was added to both sample and standard wells, mixed, and incubated for 1 h at 37 °C. After washing and removing the liquid, substrates A and B were added, followed by a stop solution. The levels of I-FABP were measured at an optical density of 450 nm using a microplate reader. All kits were obtained from MyBioSource, Inc., San Diego, CA 92195–3308.

### Histological Evaluation

Following euthanasia, the right cerebral hemisphere, ileum, and colon were dissected and fixed in 10% formaldehyde solution for 24 h, then processed into paraffin blocks. To investigate structural alterations, five µm-thick sections from all three organs were cut using a microtome and mounted on glass slides for hematoxylin and eosin (H&E) staining [25].

### Immunohistochemical Staining

The sections were mounted on ready-to-use, positively charged slides. After deparaffinization, the tissues were hydrated by gradually decreasing the alcohol concentration and then transferred to distilled water for 5 min. Antigen retrieval was performed by treating the sections with 10 mM citrate buffer (pH 6) in a microwave for 2 min, followed by a 20-min cooling period at room temperature. Sections were incubated overnight with an anti-beta-amyloid antibody (ab252816; rat monoclonal, dilution 1:100; Abcam, Cambridge, UK) and anti-malonaldehyde (anti-MDA) antibody (ab243066; mouse monoclonal, dilution 1:100; Abcam, Cambridge, UK). Next, two drops of streptavidin peroxidase were applied for 10 min, followed by two drops of biotinylated secondary antibody for 20 min. The reaction was visualized using Mayer's hematoxylin as a counterstain and diaminobenzidine (DAB) as a chromogen. Phosphate-buffered saline (PBS) was used instead of primary antibodies in the negative control sections.

### Morphometric Study

The"Leica Qwin 500C"image analyzer computer system (Leica Imaging System Ltd., Cambridge, UK) was used to obtain results in the Medical Histology and Cell Biology Department at Cairo University's Faculty of Medicine. The image analyzer was made up of an Olympus colour video camera, a colored monitor, and an IBM personal computer's hard drive that was attached to the microscope and managed by"Leica Qwin 500 C"software.

Slides were examined using a light microscope, and binary mode was employed to measure parameters in ten non-overlapping, randomly chosen high-power fields (× 400) for each section:

- Optical density of amyloid and MDA positive immunoreactivity in immunostained sections.
- Mean area percentage of GFAP positive immunoreactivity in immunostained sections.

### Statistical Methods

Data were coded and analyzed using SPSS version 28 (IBM Corp., Armonk, NY, USA) and expressed as mean ± standard deviation. Between-group comparisons were performed using one-way analysis of variance (ANOVA) followed by the Bonferroni post hoc test. Within-group comparisons between baseline and endpoint values were conducted using paired t-tests. Two-group comparisons were performed using unpaired t-tests [26]. Pearson correlation analysis was employed to assess linear relationships. A p-value < 0.05 was considered statistically significant.

## Results

### Assessment of Changes in Body Weight

**Fig. 1:**
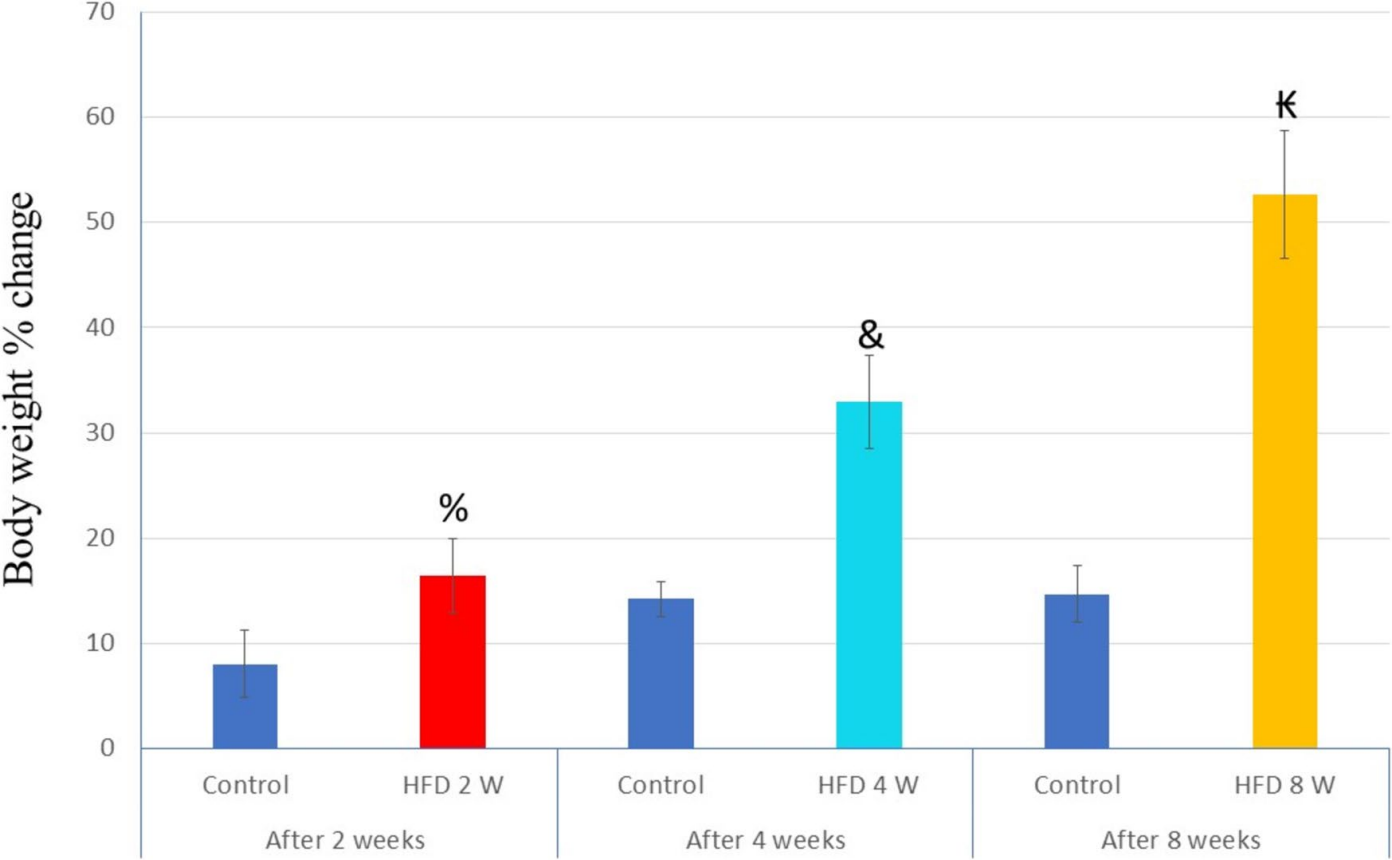
Comparison of the percent change in body weight from baseline for the HFD 2 W, HFD 4 W, and HFD 8 W groups after 2, 4, and 8 weeks, respectively, while for the control group, the comparison was between baseline and the end of the experimental period (after 8 weeks)

The initial body weights of all rats were recorded, and no significant differences were found among the groups. The mean ± SD values were as follows: control group, 174.5 ± 10.56; HFD 2 W group, 173.17 ± 6.55; HFD 4 W group, 174.83 ± 8.95; and HFD 8 W group, 175 ± 8.34 (Fig. 1) (P value > 0.05).

The time-course effect after 2, 4, and 8 weeks of normal diet intake resulted in a progressive increase in body weight in the control group, with percent changes of 8.07 ± 3.18, 19.67 ± 4.55, and 31.57 ± 7.76, respectively, compared to baseline. The percent changes in body weight for the HFD 2 W, HFD 4 W, and HFD 8 W groups were 16.44 ± 3.47%, 32.95 ± 4.39%, and 52.58 ± 6.07%, respectively, relative to their corresponding baselines.

Thus, this study demonstrated a statistically significant percentage increase in body weight in the HFD 2 W, HFD 4 W, and HFD 8 W groups compared to the control group at 2, 4, and 8 weeks, confirming the effect of HFD on body weight (P value < 0.05).

### Results of Cognitive Functions

#### Assessment of spatial memory using the Morris water maze test

The rats underwent four consecutive days of training. Baseline data for all groups showed no statistically significant differences in latency to the platform or percentage time spent in the target quadrant (Supplementary File) (P value > 0.05).

**Fig. 2:**
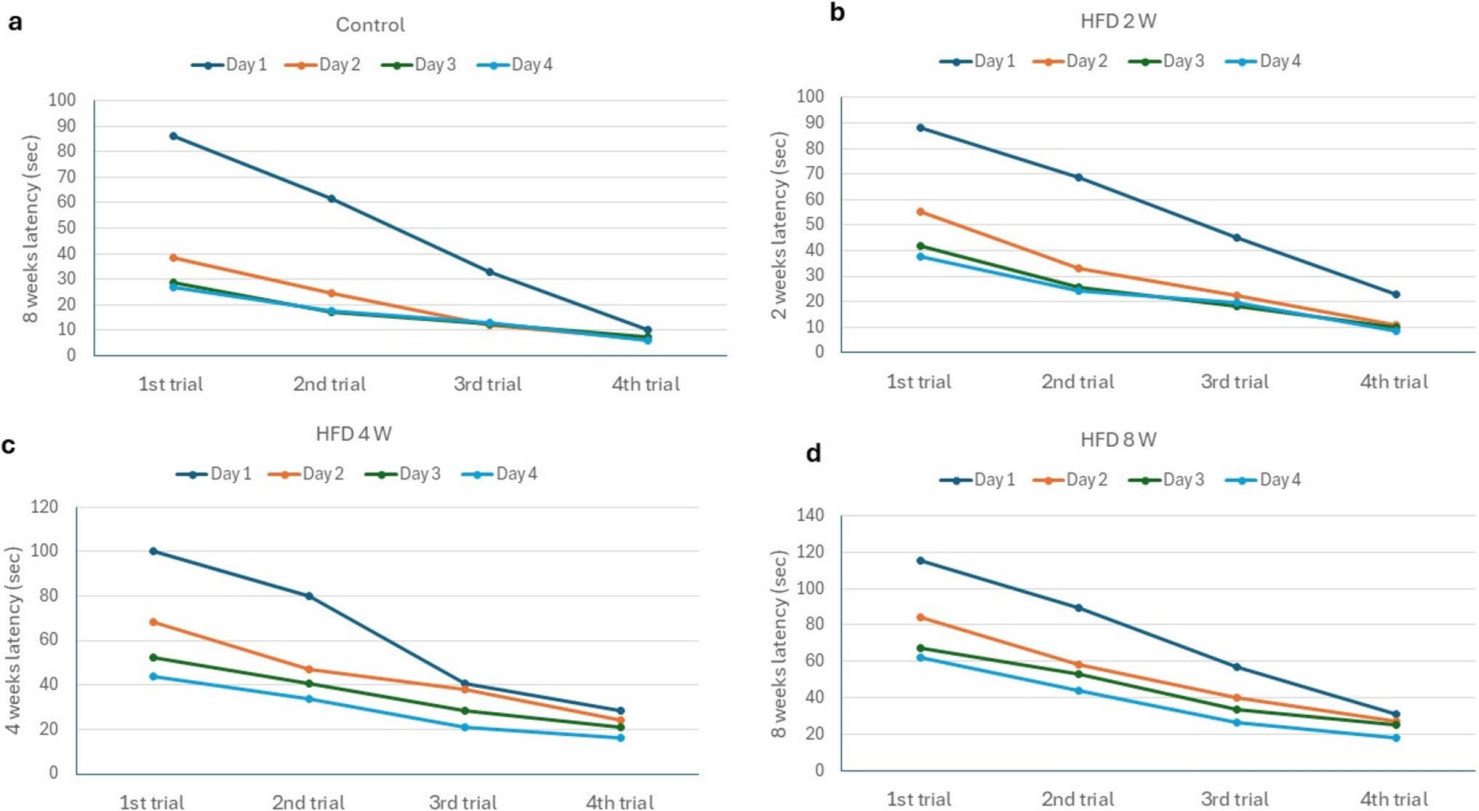
Line chart showing the mean escape latency during training for the control, HFD 2 W, HFD 4 W, and HFD 8 W groups at their respective endpoints

As shown in Fig. 2, the mean escape latency during training was influenced by the duration of HFD. On the day of the probe trial (endpoint), latency to the platform and percentage of time spent in the target quadrant during the first and second trials showed no statistical difference between the HFD 2 W and control groups (P value > 0.05). However, a significant increase in latency to the platform and a decrease in the percentage of time spent in the target quadrant during the first and second trials were observed in the HFD 4 W and HFD 8 W groups compared to both the control and HFD 2 W groups (Table 1) (P value < 0.05).

**Table 1:** The latency to the platform (seconds) and the percentage of time spent in the target quadrant for the first and second trials on the day of the probe trial

	**Control**	**HFD 2W**	**HFD 4W**	**HFD 8W**
Latency to platform (seconds) 1 st trial (endpoint)	6 ± 1.79	6.5 ± 1.87	17.17 ± 3.06*#	29 ± 6.87*#$
Percentage of time in target quadrant 1 st trial (endpoint)	71.83 ± 5	73.5 ± 9.01	51 ± 7.21*#	21.17 ± 5.63*#$
Latency to platform (seconds) 2nd trial (endpoint)	5.5 ± 1.87	7.5 ± 1.05	15.83 ± 1.72*#	27.5 ± 6.47*#$
Percentage of time in target quadrant 2nd trial (endpoint)	77.08 ± 8.99	74.08 ± 9.12	52.33 ± 3.83*#	26 ± 3.69*#$

Values are presented as mean ± SD. *: statistically significant compared to the corresponding control group value (P < 0.05), #: statistically significant compared to the corresponding HFD 2 W group value (P < 0.05), and $: statistically significant compared to the corresponding HFD 4 W group value (P < 0.05).

**Fig. 3:**
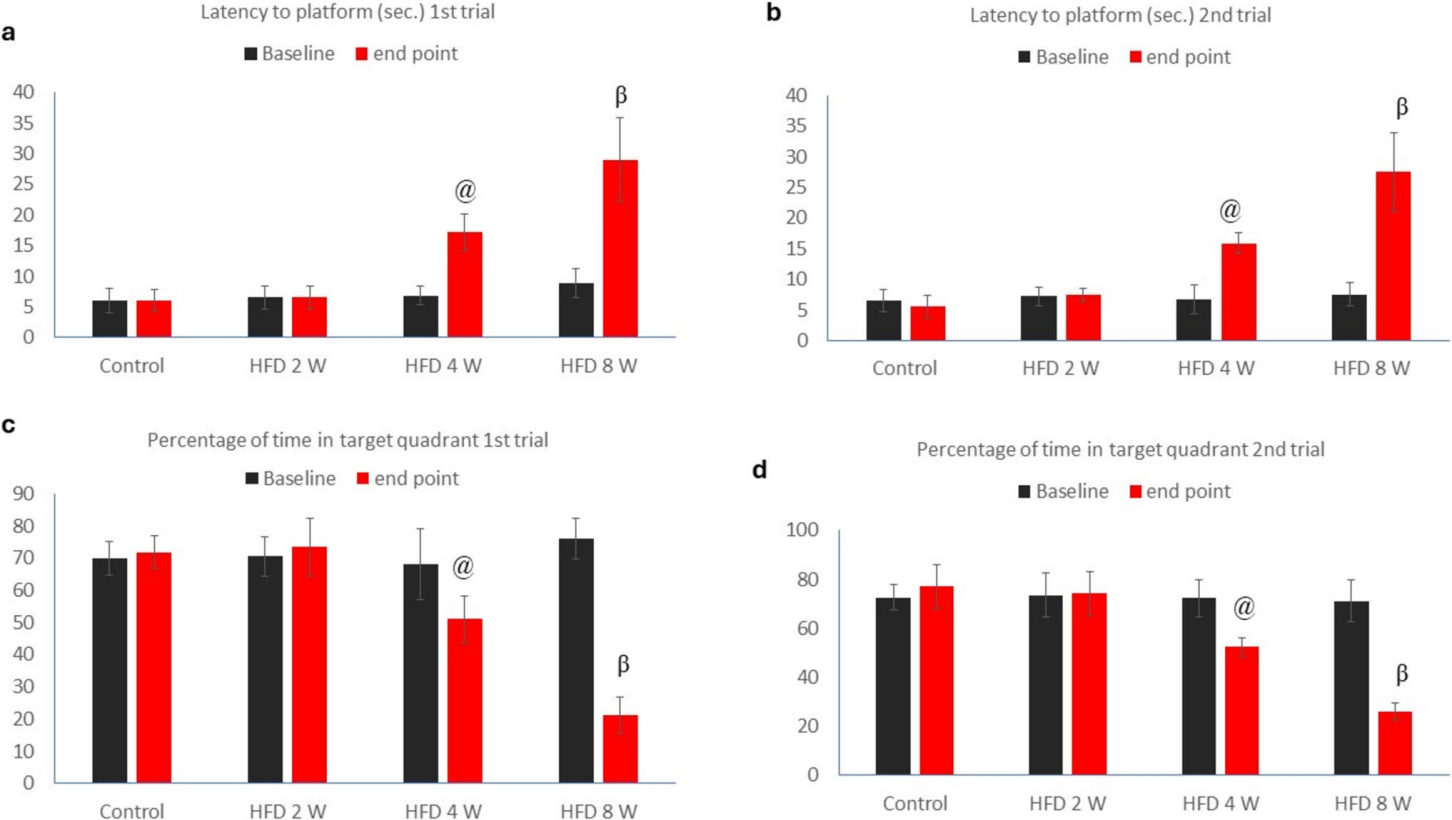
Latency to the platform (seconds) and percentage of time spent in the target quadrant during the first and second trials on the probe day. Comparisons were made between baseline and endpoint data (just before euthanasia) for each group. Values are presented as mean ± SD for baseline and endpoint data. @ denotes statistically significant differences in the HFD 4 W group compared to baseline (P < 0.05); β denotes statistically significant differences in the HFD 8 W group compared to baseline (P < 0.05)

Additionally, the current results showed no statistical difference in latency to the platform or percentage of time spent in the target quadrant during both the first and second trials between the baseline and study endpoint for the control and HFD 2 W groups (P value > 0.05). However, a significant increase in latency to the platform and a decrease in the percentage of time spent in the target quadrant were observed between baseline and endpoint in the HFD 4 W and HFD 8 W groups (Fig. 3) (P value < 0.02).

#### Assessment of Working Memory using Y-Maze and Novel Object Recognition Tests

**Fig. 4:**
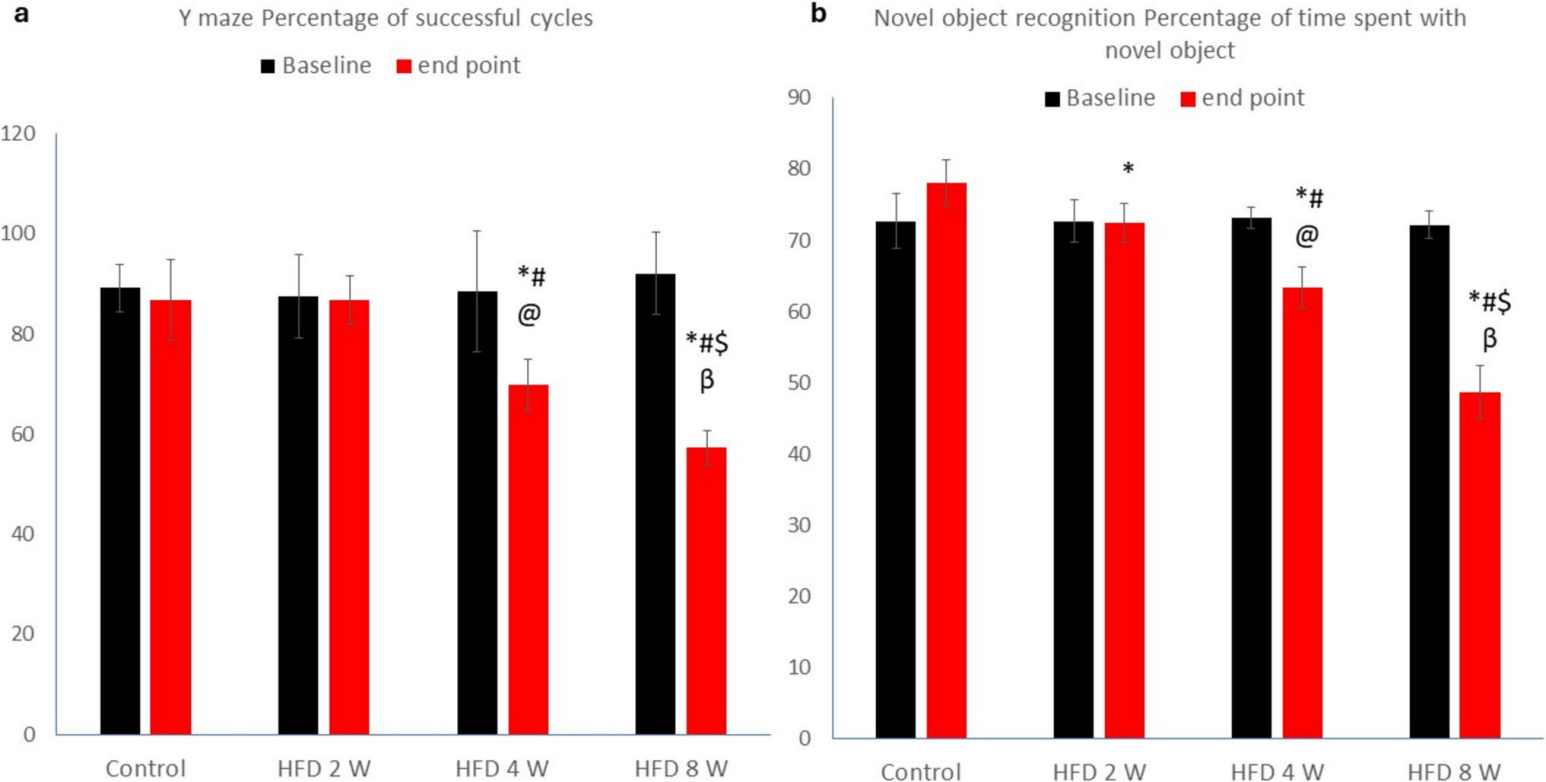
A) Percentage of successful Y-maze alternation cycles and b) percentage of time spent with the novel object at the endpoint for all groups. Values are presented as mean ± SD for baseline and endpoint data in all groups. * indicates statistically significant differences compared to the corresponding control group value (P < 0.05); # indicates statistically significant differences compared to the corresponding HFD 2 W group value (P < 0.05); $ indicates statistically significant differences compared to the corresponding HFD 4 W group value (P < 0.05); @ indicates statistically significant differences in the HFD 4 W group compared to its baseline (P < 0.05); β indicates statistically significant differences in the HFD 8 W group compared to its baseline (P < 0.05)

The baseline data for all groups showed no statistically significant differences regarding the percentage of successful cycles in the Y-maze or the percentage of time spent with the novel object (P value > 0.05). At the end of the study, the percentage of successful cycles in the Y-maze and the percentage of time spent with the novel object showed no statistical difference between the HFD 2 W and control groups. However, there was a noticeable decrease in both measures in the HFD 4 W and HFD 8 W groups compared to the control and HFD 2 W groups. Additionally, the current results showed no statistical difference between baseline and endpoint data for the Y-maze and novel object recognition tests in the HFD 2 W group (P value > 0.05). In contrast, a significant decrease was observed between baseline and endpoint results in the HFD 4 W and HFD 8 W groups (Fig. 4). (P value < 0.01).

### Biochemical Results

As presented in Table 2, all biochemical parameters exhibited a progressive and significant increase among the studied groups with increasing duration of HFD consumption. Specifically, the levels of I-FABP, LPS, TGs, and cholesterol were significantly higher in the HFD 2 W group compared to the control rats (P value < 0.05), and they were significantly elevated in the HFD 4 W group compared to both the control and HFD 2 W groups (P value < 0.05). With prolonged HFD intake, these biochemical markers continued to increase, reaching significantly higher levels in the HFD 8 W group compared to the other three groups, confirming the time-dependent effect of HFD consumption on the experimental animals (P value < 0.05).

**Table 2:** Serum levels of cholesterol, triglycerides, LPS, and I-FABP

	**Control**	**HFD 2W**	**HFD 4W**	**HFD 8W**
Triglycerides (mg/dl)	89.17 ± 7.76	127 ± 10.43*	183.17 ± 8.28*#	241.67 ± 12.91*#$
Cholesterol (mg/dl)	134.67 ± 6.59	165.83 ± 6.43*	194.5 ± 5.96*#	243.5 ± 7.77*#$
LPS (ng/ml)	117.58 ± 13.01	215.97 ± 11.41*	286.63 ± 13.56*#	551.12 ± 79.18*#$
I-FABP (ng/ml)	30.2 ± 4.14	77.37 ± 10.14*	105.52 ± 10.97*#	132.43 ± 8.03*#$

Values are presented as mean ± SD. *: statistically significant compared to the corresponding value in the control group (P < 0.05), #: statistically significant compared to the corresponding value in the HFD 2 W group (P < 0.05), and $: statistically significant compared to the corresponding value in the HFD 4 W group (P < 0.05).

### Histological Results

**Fig. 5:**
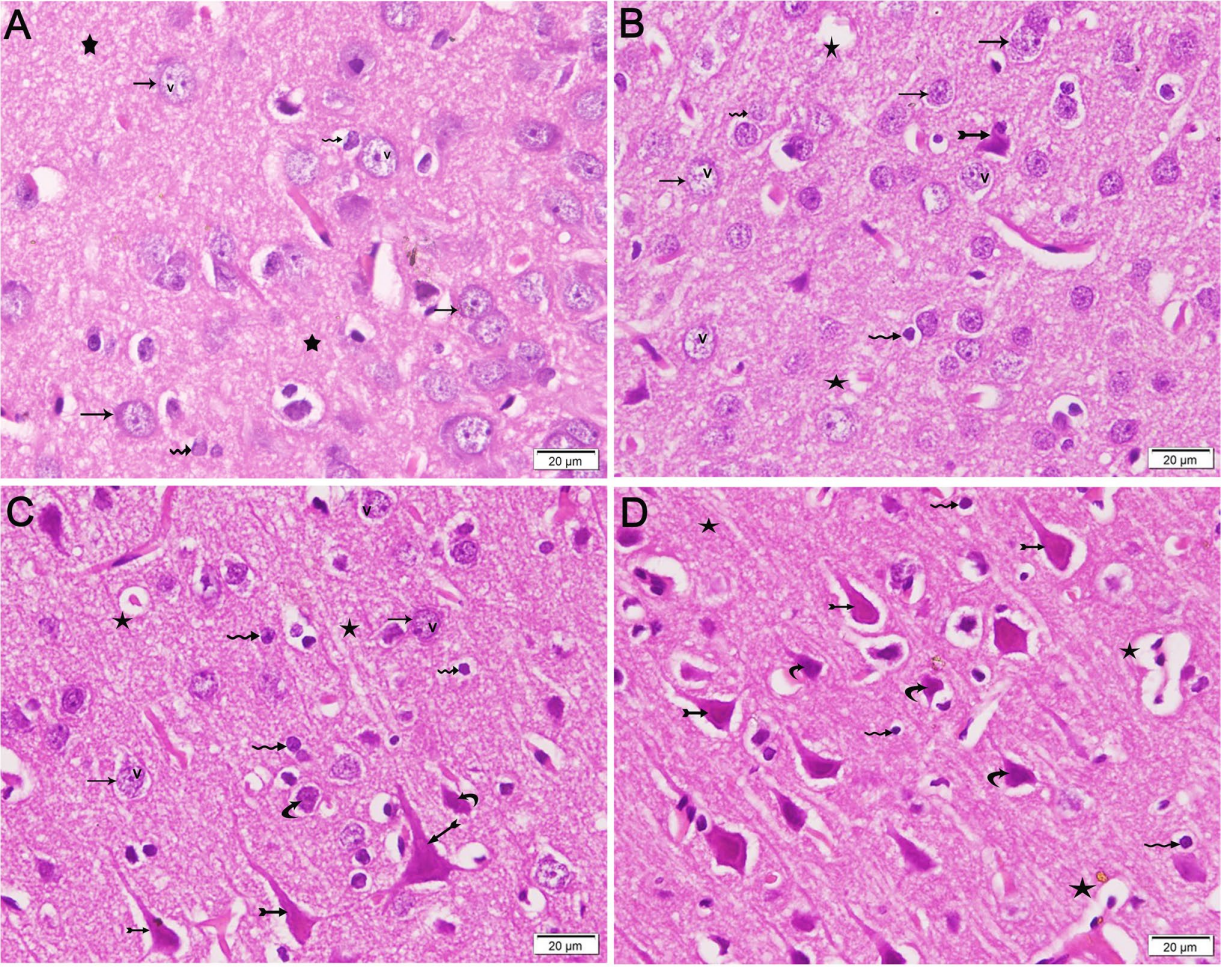
Photomicrographs of cerebral cortex sections from experimental groups (× 400). A) Control group showing large pyramidal cells with basophilic cytoplasm (arrows) and vesicular nuclei (v) surrounded by neuroglial cells (wavy arrows) in acidophilic neuropil (stars). B) HFD 2 W group showing scant degenerated pyramidal cells (bifid arrows) among large pyramidal cells (arrows) with pale nuclei (v). Vacuolations are present in the neuropil between neuroglial cells (wavy arrows). C) HFD 4 W group exhibiting few degenerated pyramidal cells (bifid arrows) surrounded by a halo, alongside large pyramidal cells (arrows) with pale nuclei (v) and neuroglial cells (wavy arrows) in acidophilic neuropil (stars). D) HFD 8 W group demonstrating degenerated pyramidal cells (bifid arrows) surrounded by a halo, shrunken cells (curved arrows), and numerous neuroglial cells with dark nuclei in vacuolated neuropil (stars)

Histological examination of brain tissues in the control group revealed large pyramidal cells with basophilic cytoplasm and vesicular nuclei associated with neuroglial cells within acidophilic neuropil (Fig. 5A). The HFD 2 W group exhibited a few degenerated pyramidal cells among the large pyramidal cells, characterized by pale nuclei (Fig. 5B). The HFD 4 W group exhibited scattered, degenerated pyramidal cells surrounded by a halo, associated with large pyramidal cells having pale nuclei and neuroglial cells in the acidophilic neuropil (Fig. 5C). The HFD 8 W group demonstrated degenerated pyramidal cells surrounded by a halo, shrunken cells, and numerous neuroglial cells with dark nuclei within vacuolated neuropil (Fig. 5D).

**Fig. 6:**
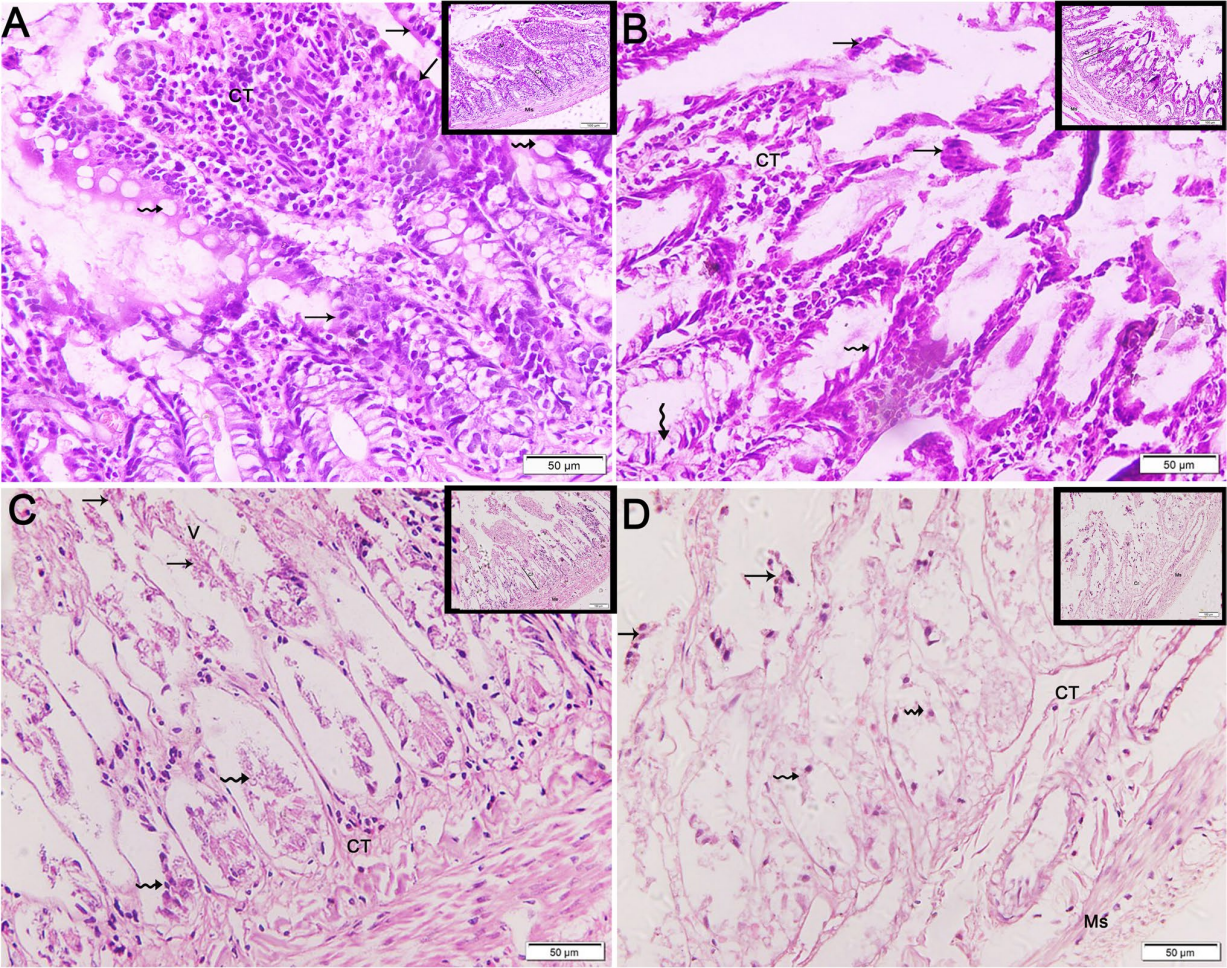
Photomicrographs of transverse sections (TS) in the ileum of experimental groups (× 200, inset × 100). A) Control group showing villi (V) and crypts (Cr) in the mucosa with the underlying smooth muscle layer (muscularis, Ms). The mucosa is lined by simple columnar cells (enterocytes, arrows) and goblet cells (wavy arrows). B) HFD 2 W group exhibiting degenerated villi (V) with preserved crypts (Cr); the underlying smooth muscles (Ms) are damaged. Surface epithelial cells (arrows) are sloughed, with many goblet cells (wavy arrows) lining the crypts. C) HFD 4 W group showing remnants of villi (V) with underlying crypts (Cr) and damaged smooth muscles (Ms). Both surface epithelial cells (arrows) and goblet cells (wavy arrows) are sloughed. D) The HFD 8 W group is exhibiting only crypts (Cr), with a complete loss of villi (V). Both surface epithelial cells (arrows) and goblet cells (wavy arrows) are sloughed. The underlying connective tissue (CT) shows vacuolations with damaged smooth muscle (muscularis, Ms)

Sections of the terminal ileum in the control group displayed the normal morphology of villi and crypts in the mucosa and the underlying smooth muscle layer. The mucosa was lined with simple columnar cells (enterocytes) and goblet cells (Fig. 6A). The HFD 2 W group showed a few degenerated villi with sloughed epithelial cells and damaged underlying smooth muscles (Fig. 6B). The HFD 4 W and HFD 8 W groups revealed remnants of villi with underlying crypts and damaged smooth muscles. Both surface epithelial cells (arrows) and goblet cells (wavy arrows) were sloughed (Figs. 6C and D).

**Fig. 7:**
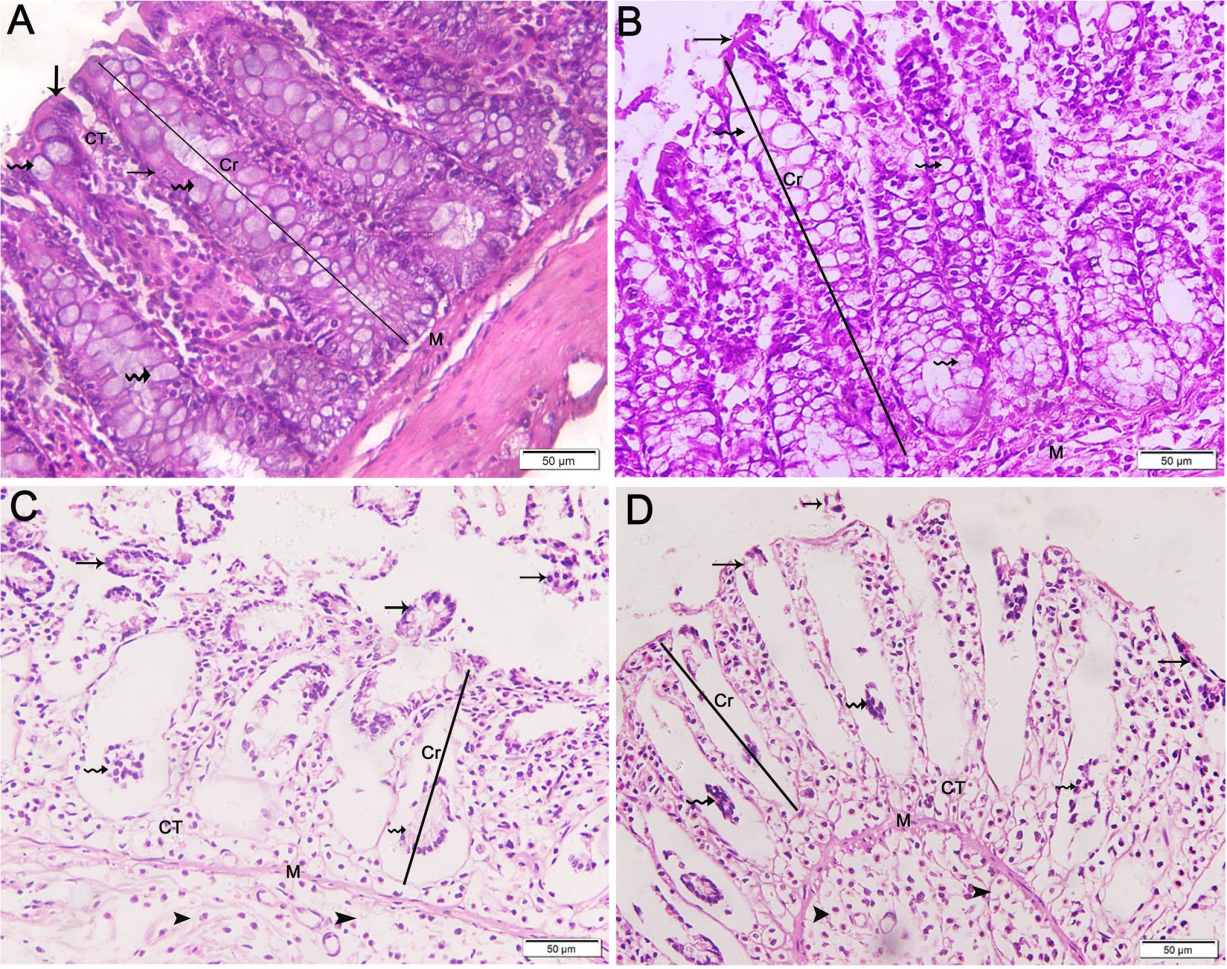
Photomicrographs of transverse sections (TS) in the colon of experimental groups (× 200). A) Control group showing mucosa composed of crypts (Cr) lined with simple columnar epithelium (arrows) and numerous goblet cells (wavy arrows). Crypts have a central connective tissue core (CT) composed of loose areolar tissue, which terminates in the muscularis mucosa (M), a layer of smooth muscle. B) The HFD 2 W group shows crypts (Cr) lined with many goblet cells (wavy arrows) and sloughing of surface epithelial cells (arrows). Vacuolations appear in the muscularis mucosa (M). C and D) HFD 4 W and HFD 8 W groups demonstrating crypts (Cr) with sloughing of surface epithelial cells (arrows) and goblet cells (wavy arrows) sloughing in the center. Connective tissue cells in the crypt centers (CT) and submucosa (arrowheads) are vacuolated with thin collagen fibers. Note the thinning of the smooth muscle layer, muscularis mucosa (M)

The colon of control animals exhibited normal mucosal histology, with crypts lined by simple columnar epithelium and numerous goblet cells (Fig. 7A). In the HFD 2 W group, crypts were lined with many goblet cells and sloughed surface epithelial cells (Fig. 7B). The HFD 4 W and HFD 8 W groups showed crypts with sloughed surface epithelial cells and goblet cells. Additionally, connective tissue cells in the crypt centers and submucosa were vacuolated with thin collagen fibers (Figs. 7C and D).

### Immunohistochemical Staining

**Fig. 8:**
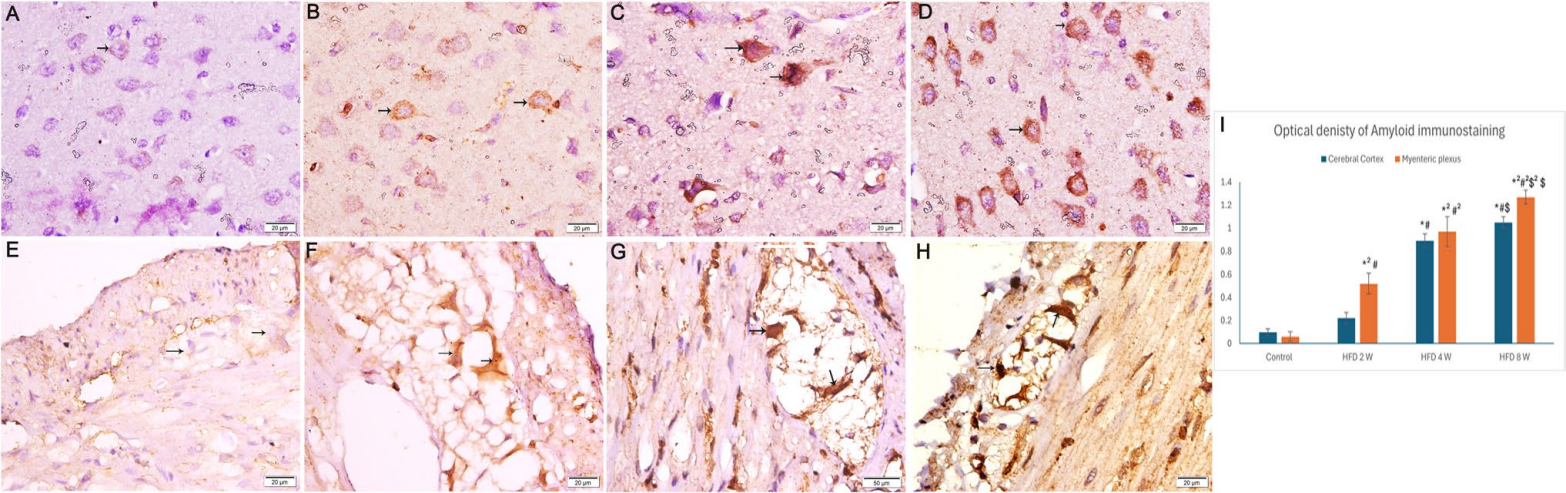
Photomicrographs of sections immunostained for amyloid β (× 400). A) Control group showing minimal immunostaining. B) HFD 2 W group exhibiting few positive pyramidal cells immunostained with amyloid β (arrows). C) The HFD 4 W group showed many positive pyramidal cells (arrows). D) HFD 8 W group exhibiting widespread amyloid β immunostaining in numerous pyramidal cells (arrows). E) The control group showed negative immunostaining in the myenteric plexus. F) HFD 2 W group revealing minimal amyloid β immunostaining in nerve cells (arrows). G) HFD 4 W group demonstrating amyloid β cytoplasmic immunostaining in many nerve cells (arrows). H) HFD 8 W group exhibiting marked cytoplasmic immunostaining in many nerve cells (arrows). I) A histogram showing the optical density of amyloid immunostaining in the cerebral cortex and myenteric plexus. *: statistically significant compared to the cerebral cortex control group (P < 0.05), *2: statistically significant compared to the myenteric plexus control group (P < 0.05), #: statistically significant compared to the cerebral cortex HFD 2 W group (P < 0.05), #2: statistically significant compared to the myenteric plexus HFD 2 W group (P < 0.05), $: statistically significant compared to the cerebral cortex HFD 4 W group (P < 0.05), $2: statistically significant compared to the myenteric plexus HFD 4 W group (P < 0.05) (A, B, C, and D: cerebral cortex; E, F, G, and H: myenteric plexus)

**Fig. 9:**
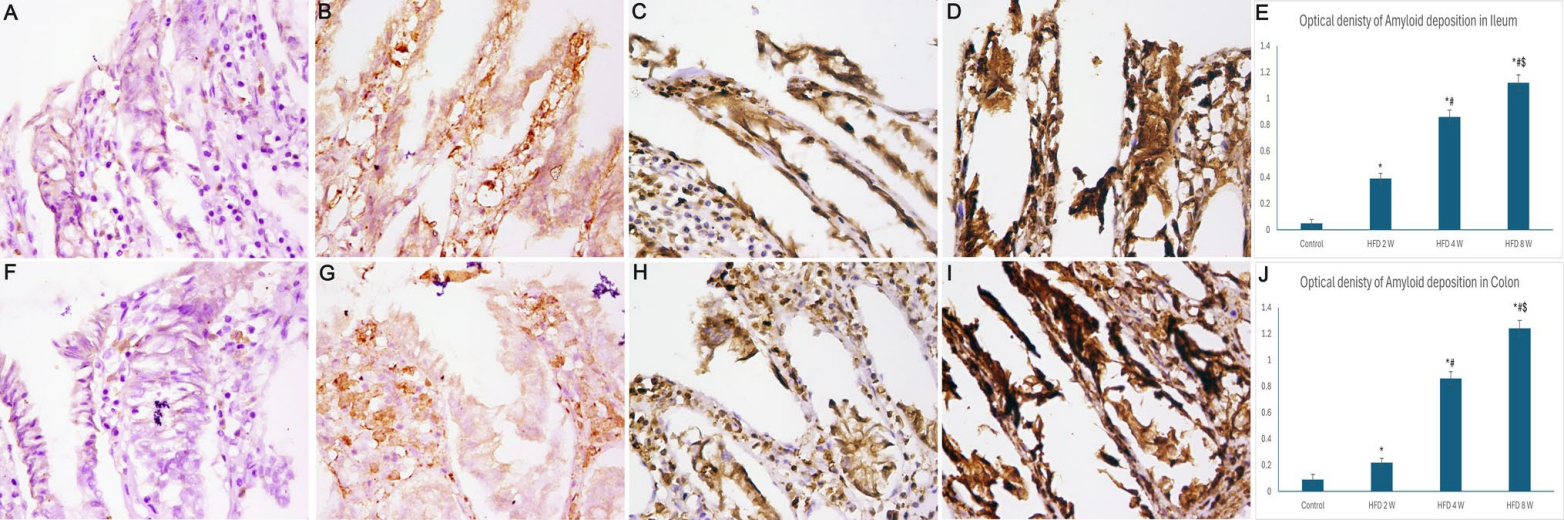
Photomicrographs of amyloid β immunostained sections (× 400). A and F) The control group exhibited faint cytoplasmic immunostaining. B and G) HFD 2 W group showing weak granular cytoplasmic amyloid β immunostaining of enterocytes. C and H) HFD 4 W group revealing moderate cytoplasmic immunostaining of enterocytes. D and I) HFD 8 W group demonstrating marked amyloid β immunostaining. E and J) Histograms showing the optical density of amyloid immunostaining in the ileum and colon. *: statistically significant compared to the control group (P < 0.05), #: statistically significant compared to the HFD 2 W group (P < 0.05), $: statistically significant compared to the HFD 4 W group (P < 0.05) (A, B, C, and D: ileum; F, G, H, and I: colon)

The control group exhibited weak immunostaining for amyloid β. In the HFD 2 W group, nerve cells in the myenteric plexus and enterocytes lining the ileum and colon showed increased amyloid β immunostaining, while the cerebral cortex exhibited pale amyloid β immunostaining. After 4 weeks of HFD, the optical density of amyloid β immunostaining was markedly increased in nerve cells of the myenteric plexus and enterocytes of the ileum and colon, along with increased immunostaining in the cerebral cortex (P value < 0.05). Furthermore, the HFD 8 W group demonstrated extensive positive amyloid β immunostaining in myenteric plexus nerve cells, enterocytes lining the ileum and colon, and the cerebral cortex (Figs. 8 and 9).

**Fig. 10:**
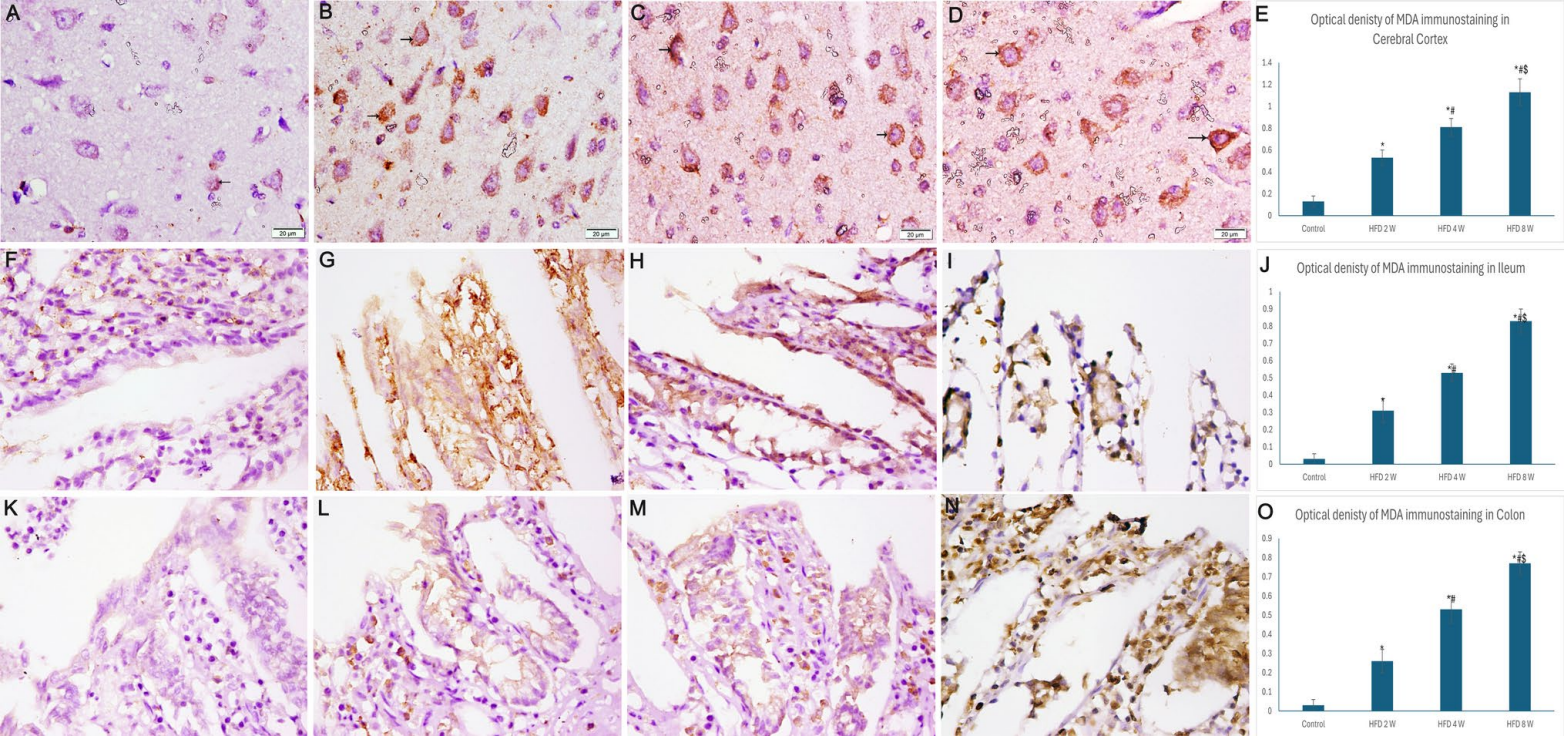
Photomicrographs of sections immunostained with MDA antibody (× 400). A, F, and K) Control group showing weak cytoplasmic immunostaining of MDA. B) HFD 2 W group revealing moderate MDA cytoplasmic immunostaining in some cells. C) The HFD 4 W group exhibited moderate immunostaining of many pyramidal cells. D) The HFD 8 W group is showing marked widespread immunostaining. G) HFD 2 W group exhibiting mild cytoplasmic immunostaining of enterocytes. H) HFD 4 W group showing moderate immunostaining of enterocytes with MDA. I) The HFD 8 W group showed increased cytoplasmic immunostaining. K) HFD 2 W group revealing weak cytoplasmic immunostaining of MDA. L) HFD 4 W group exhibiting increased cytoplasmic immunostaining of enterocytes. M) The HFD 8 W group showed intense immunostaining of enterocytes. E, J, and O) Histograms showing the optical density of MDA immunostaining. *: statistically significant compared to the control group (P < 0.05), #: statistically significant compared to the HFD 2 W group (P < 0.05), $: statistically significant compared to the HFD 4 W group (P < 0.05) (A, B, C, and D: cerebral cortex; F, G, H, and I: ileum; K, L, M, and N: colon)

Control rats showed weak granular cytoplasmic immunostaining of MDA in surface epithelial cells of the colon and ileum, as well as pyramidal cells of the cerebral cortex. After 2 weeks of HFD consumption, moderate MDA immunostaining appeared in the cytoplasm, which markedly increased after 4 weeks of HFD (P value < 0.05). The mean area percentage of MDA immunostaining in the colon, ileum, and cerebral cortex was significantly increased in HFD groups compared to controls (P value < 0.05) (Fig. 10).

**Fig. 11:**
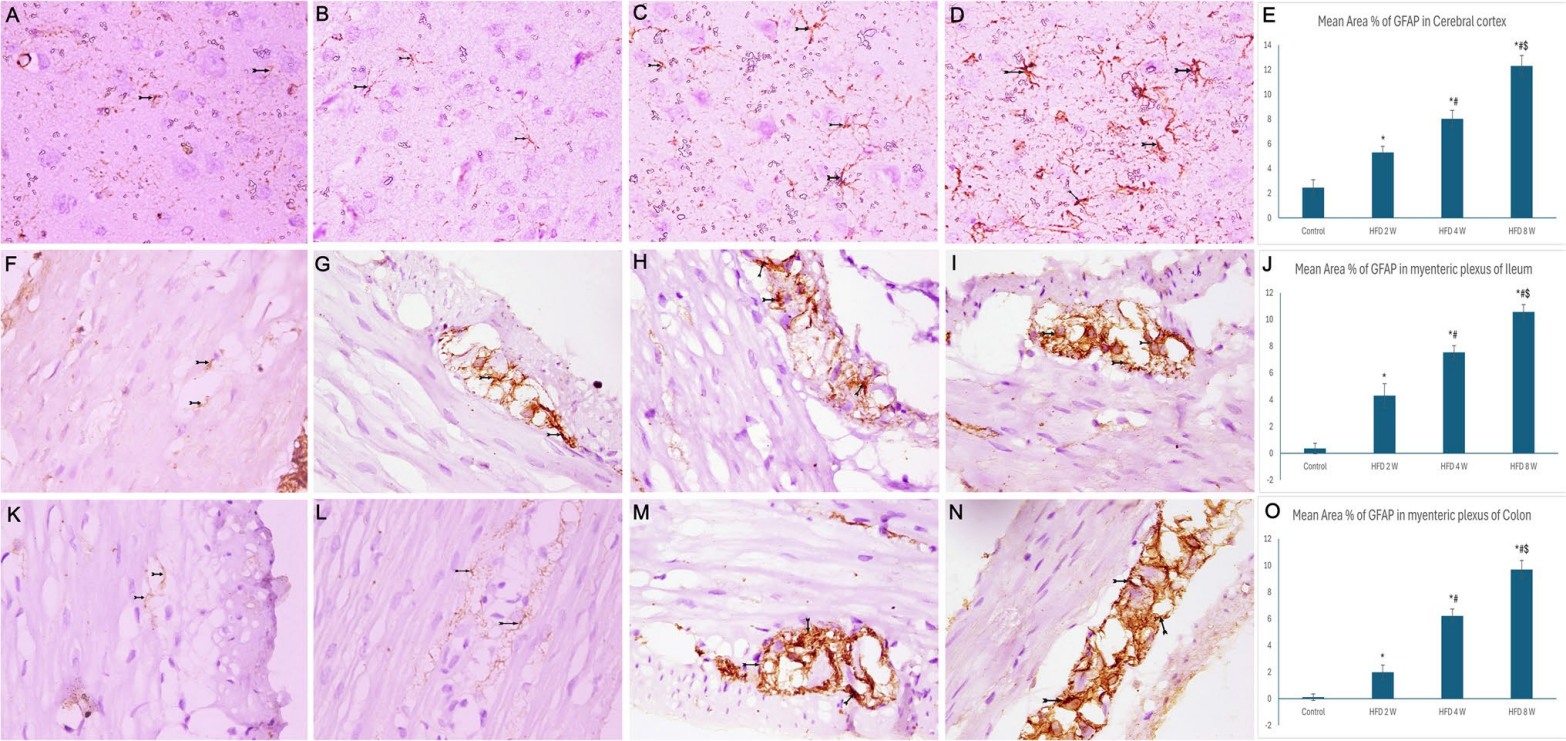
Photomicrographs of sections immunostained with GFAP antibody (× 400). A, F, and K) The control group revealed a few astrocytes (bifid arrows) that were immunostained by GFAP. B, G, and L) HFD 2 W group showing some astrocytes (bifid arrows) immunostained by GFAP. C, H, and M) HFD 4 W group demonstrating many GFAP-immunostained astrocytes. D, I, and N) HFD 8 W group exhibiting widespread astrocyte immunostaining by GFAP. E, J, and O) Histograms showing the mean area percentage of GFAP immunostaining. *: statistically significant compared to the control group (P < 0.05), #: statistically significant compared to the HFD 2 W group (P < 0.05), $: statistically significant compared to the HFD 4 W group (P < 0.05) (A, B, C, and D: cerebral cortex; F, G, H, and I: ileum; K, L, M, and N: colon)

The mean area percentage of astrocytes immunostained with GFAP increased between nerve cells of the myenteric plexus and in the cerebral cortex of the HFD 2 W group compared to controls (P value < 0.05). The HFD 4 W group exhibited numerous astrocytes immunostained with GFAP, with a significant further increase in the HFD 8 W group compared to the other three groups (P value < 0.05) (Fig. 11).

## Correlations

**Fig. 12:**
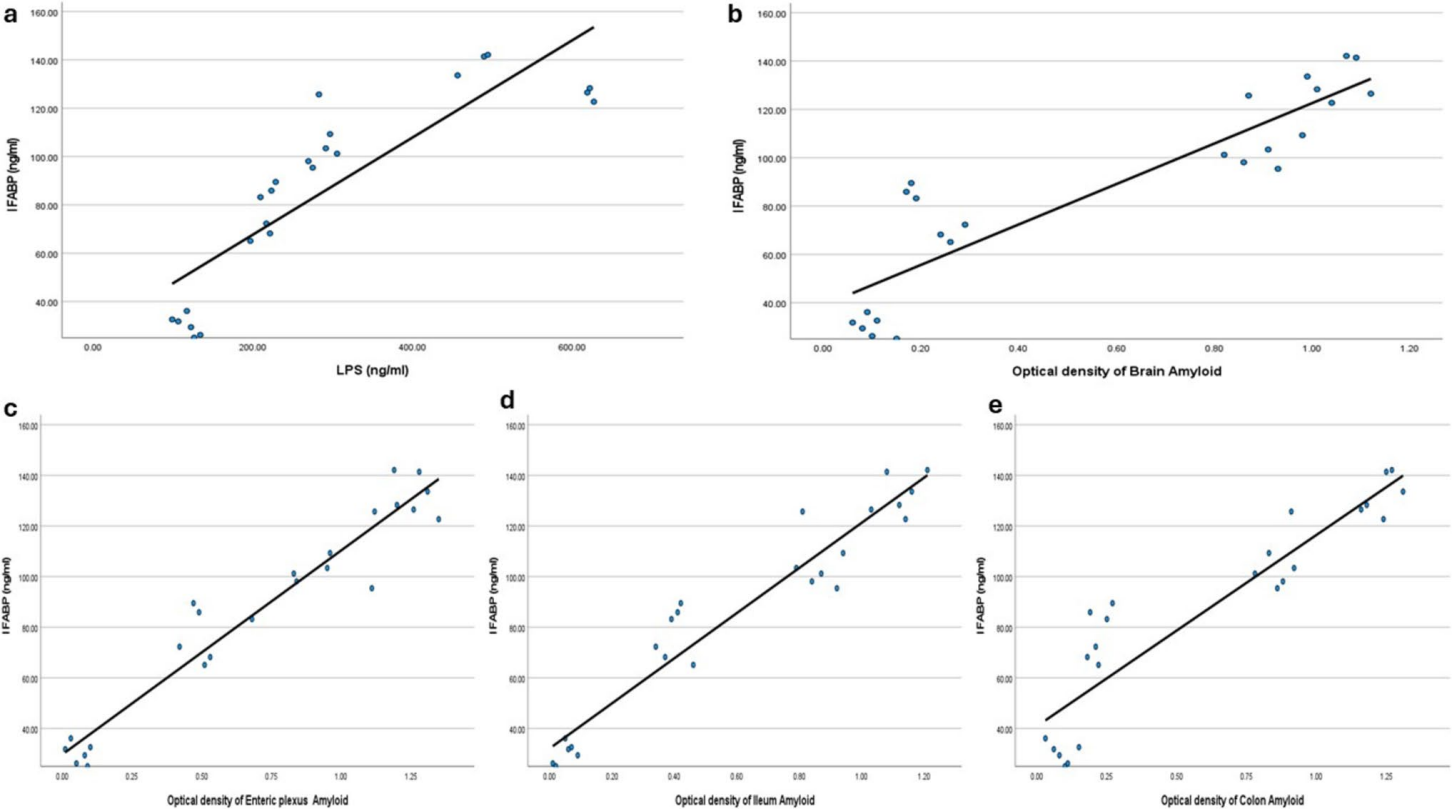
A, b, c, d, and e) Positive correlations between I-FABP and the levels of LPS (r = 0.859, P < 0.001), the optical density of brain amyloid (r = 0.899, P < 0.001), the optical density of enteric plexus amyloid (r = 0.966, P < 0.001), the optical density of amyloid deposition in the ileum wall (r = 0.961, P < 0.001), and the optical density of amyloid in the colon wall (r = 0.919, P < 0.001)

The current results showed a positive correlation between I-FABP and each of the following: (Fig. 12).

- Levels of LPS (r = 0.859, P < 0.001)
- The optical density of brain amyloid (r = 0.899, P < 0.001)
- The optical density of enteric plexus amyloid (r = 0.966, P < 0.001)
- The optical density of amyloid deposition in the ileum wall (r = 0.961, P < 0.001)
- The optical density of amyloid in the colon wall (r = 0.919, P < 0.001).

## Discussion

Long-term intake of HFD and obesity can alter cognitive functions and induce brain damage [27]. Previous literature has highlighted the link between HFD and the development of peripheral tissue inflammation, as well as its role in mediating neuroinflammation and neurodegeneration [28]. In this context, Zhao et al. reported that HFD intake can exacerbate inflammatory cellular infiltration into the colon and may even induce a phenotype resembling ulcerative colitis [29]. Previous research by Zhuang et al. investigated the effects of long-term HFD consumption for 6 and 9 months on the brain function of adult male C57BL/6 J mice [30]. Moreover, feeding apolipoprotein E-deficient mice a 21% HFD for 8 weeks resulted in impaired cognitive function, as assessed using the novel object recognition test and the Y-maze test [31].

Baym et al. reported that exposure to HFD significantly impacts the hippocampus in both young and adult animals [32]. The hippocampus is vital for most learning and memory processes, and abnormalities in this region can be detected even in the early stages of neurodegenerative dementias, including vascular dementia and AD [33]. Although rodent models have revealed that HFD can induce hippocampal-dependent memory impairment after long-term intake (more than 4 weeks) [34–36], few studies have assessed the consequences of short-term (up to 4 weeks) HFD feeding. These studies reported only isolated brain effects [37–39]. Thus, the question arises: how quickly does a shift to HFD affect hippocampal functions and structure? Additionally, the impact of short-term HFD intake on gut integrity and permeability remains to be investigated. Furthermore, the time-course effect of HFD intake on the local ENS and brain gliosis has not been examined.

Currently, we found that HFD intake for 2 weeks did not significantly affect cognitive functions. At the tissue level, histological changes in the intestinal mucosa revealed few degenerated villi with sloughed epithelial cells, whereas brain tissues exhibited scant degeneration of pyramidal cells among large pyramidal cells with pale nuclei. With an increasing duration of 60% HFD feeding in the HFD 4 W and 8 W groups, histological changes became more pronounced, and cognitive functions declined. The current results documented altered cognitive functions 4 weeks after continuous HFD intake, supported by increased latency to the platform and decreased percentage of time spent in the target quadrant for the 1 st and 2nd trials of the MWM test, as well as decreased percentage of successful cycles in the Y-maze test and reduced time spent with the novel object.

In the present study, 2 weeks of HFD intake significantly increased I-FABP and LPS compared to control animals. Gut dysbiosis and impaired intestinal barrier function can induce a leaky gut, allowing gut antigens to enter the circulation and triggering both local and systemic inflammatory changes, a phenomenon known as"metabolic endotoxemia"[40]. In this context, Wan et al. detected an increased number of LPS-producing bacteria secondary to HFD intake [41]. The proinflammatory mediator LPS, produced by Gram-negative bacteria, can disrupt the integrity of the tight junction-mediated intestinal barrier, leading to gut barrier injury [42, 43]. Through the damaged barrier, bacteria-derived molecules and other antigens can enter the circulation [44]. Our findings revealed mucosal damage in the ileum and colon with HFD intake, where villi length decreased, and enterocyte sloughing significantly increased in the HFD 8 W group compared to both the HFD 2 W and 4 W groups. Another study demonstrated that HFD-fed animals showed decreased colon length, crypt depth, and villi length in the small intestine [45].

The primary components of the neuronal cytoskeleton are neurofilaments and α-internexin. Deficiencies in neurofilaments have been linked to neuronal loss and neurodegenerative disorders, explaining the nerve cell damage and neuronal dysfunction observed in the study by Ogata et al. [46]. Additionally, Alkan et al. reported irregularly shaped neurons in the HFD group, exhibiting degeneration and reduced quantity compared to controls [47]. These results support the hypothesis that rats on the HFD have reduced plasticity, which explains the pyramidal cell damage observed in the cerebral cortex in our study.

Besides promoting systemic inflammation, LPS leads to progressive neuroinflammation and induces neurodegeneration [48]. LPS can promote ROS release and selectively enhance tumor necrosis factor-alpha release, which may promote amyloid plaque formation [49]. Consistent with our results, I-FABP levels showed a positive correlation with LPS levels and with the optical density of amyloid β deposition in the brain and enteric plexus. Furthermore, Tan and Norhaizan stated that consuming HFD products increases the risk of chronic inflammation and activates multiple downstream signaling pathways [50], ultimately leading to increased oxidative stress and disturbances in lipid metabolism. Additionally, this was demonstrated by Yang et al., who showed that HFD markedly decreased hepatic superoxide dismutase (SOD), glutathione peroxidase (GSH-Px), and catalase (CAT) levels while increasing hepatic lipid peroxidation and MDA levels [51]. In our study, the optical density of MDA was markedly increased in the cerebral cortex, ileum, and colon.

In this study, we chose Sprague–Dawley (SD) rats. A previous study by Marques and colleagues (2015) reported that intake of 45% HFD induces earlier and more pronounced metabolic changes in Wistar rats than in SD rats, which may shift the current model toward metabolic syndrome with insulin resistance if Wistar rats were used instead of SD rats [52]. Additionally, SD rats exhibit diet behavior and food intake patterns similar to humans, making them a suitable model for studying diet-induced obesity and alterations in lipid metabolism [53].

Maintenance of energy homeostasis requires appropriate integration among multiple central neurocircuits, which involves inputs from the enteric plexus and the vagus nerve. Disruption of any of these neural inputs may precipitate the onset and progression of obesity [54]. This may explain the percentage change in weight gain after 2, 4, and 8 weeks of HFD intake compared to the corresponding time points in the control group. Moreover, long-term HFD consumption has been associated with the loss of myenteric neurons, particularly those linked to disrupted gastrointestinal motility and gastric emptying [55, 56].

In AD animal models fed with HFD, previous research has revealed enhanced amyloid burden, increased hyperphosphorylated tau protein levels, and more severe inflammation [57, 58]. These findings are consistent with ours, where the optical density of amyloid immunohistostaining significantly increased with longer durations of HFD intake. The occurrence of myenteric neurodegeneration and amyloid β deposition after HFD intake remains unclear. In this context, we investigated amyloid β deposition locally in the myenteric plexus and its relationship to the onset of amyloid β deposition in the brain tissues of HFD-fed rats. Notably, the optical density of amyloid immunostaining increased in myenteric plexus before it did in the cerebral cortex. Recent research by Liu et al. revealed strong intracellular amyloid β (Aβ42) deposition in the colons of young APP/PS1 mice, although their brains exhibited no evident Aβ42 immunoreactivity [9]. Additionally, they found that enteric neurons of AD animals exhibited significantly higher intracellular Aβ40 and Aβ42 immunoreactivity compared to controls. In other words, cases with AD brains may experience intestinal dysfunction and ENS damage before typical clinical symptoms appear.

Beyond amyloid β and tau proteins, recent studies have highlighted other proteins as markers for AD, such as brain-derived neurotrophic factor and glial fibrillary acidic protein (GFAP) [59]. The ENS contains astroglia cells similar to those in the CNS, which are stained by the glial cell marker GFAP. The number of astroglia cells, particularly microglia and astrocytes, is linked to AD pathogenesis [60]. Reactive astrocytes contribute to the neuroinflammation associated with AD by releasing various cytokines, inflammatory biomarkers, and ROS [61].

Astrocytes are the most prevalent cells in the CNS and dynamically contribute to maintaining normal neuronal processes through various supportive functions. Accordingly, a growing body of research has linked the physiological or pathological regulation of body energy balance and metabolic processes to astrocyte function. Recent studies indicate that HFD induces neuroinflammation and reactive gliosis in the hypothalamus prior to noticeable body weight gain [62–64]. In the current study, we demonstrated the normal appearance of astrocytes in the cerebral cortex of control rats, with a significant increase in their number after 2 weeks on HFD. The increase in the area percentage of GFAP immunostaining in the enteric plexus, which preceded their increase in brain tissues, suggests an earlier rise in enteric plexus astrocytes before their increase in the cerebral cortex. Additionally, previous research has reported that reactive astrocytes can appear before early pathological hallmarks of AD, such as Aβ and tau, during disease progression [59]. Furthermore, Li et al. stated that glial activation and persistent neuroinflammatory alterations are common pathogenic features of AD linked to synaptic stripping and neuronal death [65].

Despite the interesting findings of our study, there are limitations, including a lack of direct assessment of the mechanistic pathways that potentially explain the gut-brain link. Moreover, the small sample size of six rats per group, the use of a single control group across multiple time points, and the unavailability of software analysis for the MWM test may confound the interpretation of HFD-mediated changes. Therefore, future research should focus on defining and precisely elucidating the underlying mechanisms and time courses linking HFD consumption to CNS and intestinal disorders, as well as providing more detailed analyses of glial subtypes.

## Conclusion

To our knowledge, this is one of the first studies to evaluate the impact of 60% HFD intake over a short period. Our findings suggest that increasing the duration of 60% HFD consumption in rats was associated with a progressive increase in body weight, marked impairment of cognitive functions manifested as progressive deterioration of spatial and working memory, and a significant progressive increase in lipid profile levels as well as amyloid deposition in ENS neurons, specifically the myenteric plexus neurons. Notably, the changes observed in the ENS preceded cortical involvement. Future studies should focus on delineating the temporal progression and underlying mechanisms linking HFD intake to enteric and CNS dysfunctions.

## Supplementary Information

Below is the link to the electronic supplementary material.

Supplementary file1 (DOCX 19 KB)

## Data Availability

No datasets were generated or analysed during the current study.
