# Supplementary material for: Interruption of the Gut Integrity Contributes to Early Accumulation of Amyloid-β in the Enteric Nervous System in Rats Supplemented with a High-Fat Diet
Source: Mol Neurobiol. 2025 Aug 8;62(12):16472–88. doi: 10.1007/s12035-025-05261-1 (PMC12559146; doi:10.1007/s12035-025-05261-1)
Supplement: Supplementary file 1 — Supplementary file1 (DOCX 19 KB) [file 12035_2025_5261_MOESM1_ESM.docx]

**Composition of control diet (10% *kcal% Fat*) for the control group: D12450Ji, Research Diets**

| **Class description** | **Ingredients** | **Grams** |
| --- | --- | --- |
| Protein | Casein, Lactic, 30 Mesh | 200.00 g |
| Protein | Cystine, L | 3.00 g |
| Carbohydrate | Starch, Corn | 506.20 g |
| Carbohydrate | Lodex 10 | 125.00 g |
| Carbohydrate | Sucrose, Fine Granulated | 72.80 g |
| Fiber | Solka Floc, FCC200 | 50.00 g |
| Fat | Soybean Oil, USP | 25.00 g |
| Fat | Lard | 20.00 g |
| Mineral | [S10026B](https://www.researchdiets.com/formulas/S10026B) | 50.00 g |
| Vitamin | Choline Bitartrate | 2.00 g |
| Vitamin | [V10001C](https://www.researchdiets.com/formulas/V10001C) | 1.00 g |
| Dye | Dye, Yellow FD&C #5, Alum. Lake 35-42% | 0.04 g |
| Dye | Dye, Blue FD&C #1, Alum. Lake 35-42% | 0.01 g |
|  | Total: | 1055.05 g |

| **Class description** | **Ingredients** | **Grams** |
| --- | --- | --- |
| Protein | Casein, Lactic, 30 Mesh | 200.00 g |
| Protein | Cystine, L | 3.00 g |
| Carbohydrate | Lodex 10 | 125.00 g |
| Carbohydrate | Sucrose, Fine Granulated | 72.80 g |
| Fiber | Solka Floc, FCC200 | 50.00 g |
| Fat | Lard | 245.00 g |
| Fat | Soybean Oil, USP | 25.00 g |
| Mineral | [S10026B](https://researchdiets.com/formulas/S10026B) | 50.00 g |
| Vitamin | Choline Bitartrate | 2.00 g |
| Vitamin | [V10001C](https://researchdiets.com/formulas/V10001C) | 1.00 g |
| Dye | Dye, Blue FD&C #1, Alum. Lake 35-42% | 0.05 g |
|  | Total: | 773.85 g |

**Composition of high fat diet (60 *kcal% Fat*) : Research Diets D12492**

**Baseline data for all groups. Latency to the platform (sec.), percentage of time spent in the target quadrant for 1st and 2nd trials of the day of probing, Y-maze, and novel object recognition tests**

|  | **Control** | **HFD 2 W** | **HFD 4 W** | **HFD 8 W** |
| --- | --- | --- | --- | --- |
| **Latency to platform (sec.) 1st trial (Baseline)** | 6±2 | 6.5±1.87 | 6.83±1.47 | 8.83±2.32 |
| **Percentage of time in target quadrant 1st trial (Baseline)** | 69.83±5.23 | 70.5±6.19 | 68.17±10.98 | 76±6.26 |
| **Latency to platform (sec.) 2nd trial (Baseline)** | 6.5±1.87 | 7.17±1.47 | 6.67±2.42 | 7.5±1.87 |
| **Percentage of time in target quadrant 2nd trial (Baseline)** | 72.5±5.13 | 73.5±9.01 | 72.17±7.44 | 71.17±8.47 |
| **Y maze Percentage of successful cycles (Baseline)** | 89.17±4.71 | 87.5±8.26 | 88.5±12.16 | 92±8.22 |
| **Novel object recognition Percentage of time spent with novel object (Baseline)** | 72.67±3.83 | 72.67±2.93 | 73.08±1.5 | 72.17±1.94 |
